# Hot Water and Soap in Ptyalism

**Published:** 1853-08

**Authors:** 


From the Southern Journal.

*Hot Water and Soap in Ptyalism.*

A great variety of remedies have, from time to time, been employed in the treatment of ptyalism; every practitioner having his own favorite remedy. Tar water, solution of creosote, lead water, sumach root tea, sage tea and honey, alum, spts. turpentine, &c., have each acquired more or less reputation in the hands of different practitioners; but we have never been satisfied with any of these remedies, though we have repeatedly prescribed them. Very recently, having to treat a severe case of accidental ptyalism, we prescribed a *hot* solution of soap. The patient was suffering with severe pain of the gums and copious salivary discharge—a few drams of spirits of soap was added to one pint of *hot* water, and the patient directed to take it into the mouth, as hot as he could bear, and retain it until the surplus heat was exhausted, and repeat for an hour, allowing an interval of half an hour for rest. At the end of twelve hours, we had the gratification to find the patient almost entirely relieved of the pain—the swelling and redness of the gums and soft parts about the mouth rapidly diminished, and in a few days, by the persevering use of the hot water, the patient was free of all uneasiness about the mouth.

The value of hot water was suggested from having observed the good effects of hot tar water in a similar case; the patient, a delicate, nervous female, was directed to use warm tar water occasionally, but finding that the hotter the water, the greater relief was afforded, she continued using it as hot as the mouth could bear it. We had noticed too, the effects of the prolonged immersion of the hands of washwomen in warm *soap suds*, corrugating and puckering the skin of the hands and fingers to such a degree that the blood seemed almost expelled from the vessels of the part.

The first effect of hot water in mercurial sore mouth, seems to be relief from the painful distension of the soft parts, and secondly, an anemic condition of the blood vessels from contraction or collapse of the capillaries. The stronger preparations of soap are powerfully astringent—the kind used in preparing the spts. sapo. was the castile—it may be that *turpentine* soap is preferable.

